# Low serum level and chronic toxicity for methotrexate. Case Report

**DOI:** 10.5339/qmj.2023.31

**Published:** 2023-11-13

**Authors:** Ibtisam Musameh, Faten Al-Bakri, Asmaa Ezzeldin

**Affiliations:** ^1^Emergency Department, Hamad General Hospital, Qatar E-mail: IMusameh@hamad.qa; ^2^Clinical Pharmacy Service, Hamad General Hospital, Qatar

**Keywords:** methotrexate, overdose, toxicity, serum level, case reports, leucovorin

## Abstract

Introduction: Methotrexate (MTX) is a folic acid antagonist used to treat different immunological or proliferative illnesses because of its anti-proliferative and anti-inflammatory effects. MTX Toxicity is considered a severe problem. Although acute toxicity related to high-dose administration (doses ≥500 mg/m^2^) can be predicted based on the given dose, chronic toxicity still has no specific factors to predict it, so treatment depends on the history and symptoms of toxicity. MTX was initially used for oncology indications with high cyclic doses, then expanded to non-oncology indications with different low doses and frequencies. This significant change in doses resulted in dosing errors that contributed to MTX toxicity reports. Measures to prevent the toxicity of MTX should be implemented.

Case: A 66-year-old female patient ingested 10 mg of MTX daily for one month instead of the once-toxicity symptoms. The serum level of MTX was requested, and treatment with folinic acid was initiated until the patient improved with the discontinuation of MTX.

Discussion: There is limited literature about the lack the total cumulative dose, duration of intake, or serum level of MTX. All this information was provided in this case report, but drug-drug interactions were not reviewed, although aspirin and pantoprazole were identified as having interactions with methotrexate in this patient. Minimum total cumulative dose identification may help assess the toxicity risk in such patients.

Conclusion: Low-dose MTX chronic toxicity still needs further information to guide the patient’s risk of toxicity and when to initiate treatment. Safety-practical measures should be implemented to prevent such administration errors.

## Introduction

Methotrexate is a folate antimetabolite that inhibits DNA synthesis, repair, and cellular replication. Methotrexate has been used in multiple oncology and non-oncology indications (e.g., dermatology, ectopic pregnancy, rheumatoid arthritis, etc.).^1^ The doses for each indication are dramatically different. High doses of methotrexate (HDMTX) > 500 mg/m^2^ are used in oncology indications based on other tumor protocols. Treatment response, side effects, and toxicity from HDMTX vary between patients depending on different factors, even if the same dose is administered.2 Therapeutic drug monitoring (TDM) of MTX is recommended with HDMTX. TDM standards vary significantly between tumor types and institutes of medicine. Any delay in the MTX level decline according to the used protocol means a delay in MTX elimination, which will increase the risk of side effects and toxicity of MTX. Nevertheless, toxicity and side effects are unrelated to TDM levels.^3,4^ MTX is the first choice in treating non-oncology indications, like rheumatoid arthritis, as low once-weekly doses.^5^ TDM is not recommended for non-oncology indications as no standard level is available for chronic low doses of MTX due to its pharmacokinetic properties. Toxicity in chronic low doses is assessed based on history, signs, and symptoms.^6^ Methotrexate toxicity can be a result of repeated oral overmedication or high-dose intrathecal, intravenous, and intramuscular methotrexate administration, either accidentally or intentionally.^7,8,9^ One study reported dosing errors in five out of 22 cases.^10^ Another study assessing patient adherence to MTX found that many rheumatoid arthritis patients do not take MTX as directed.^11^

In a cross-sectional investigation comparing acute and chronic MTX toxicity, mucosal ulcers, which occurred in 92.8 percent of cases of chronic MTX toxicity, were the most often reported symptom.

This was followed by skin lesions (42%), nausea and vomiting (35%), diarrhea (28%), and stomach pain (28%). While the most frequent toxicity associated with acute high doses of MTX is acute renal damage (2%–12% of patients), gastrointestinal toxicity (0%–30%), liver toxicity (25–60%), and neurotoxicity (up to 15% of patients), these are only a few of the significant adverse reactions that HDMTX can cause, even though it is generally safe to deliver to most patients.^6,12^ The continuous reporting of dosing errors with methotrexate indicates that more measures are still required to prevent this type of error. To publish this case report of methotrexate overdose, permission was received from the Hamad Medical Corporation Medical Research Center (MRC-04-23-271).

## Case Presentation

A 66-year-old female patient weighing 70 kg had a history of diabetes, hypertension, hypothyroidism, rheumatoid arthritis, and a pancreatic neuroendocrine tumor with liver metastasis. She had a distal pancreatectomy, splenectomy, vascular reconstruction, adhesiolysis, and atypical liver resection of segments 3, 4a, and 4b, as well as a cholecystectomy.

During the patient’s last appointment at the rheumatology clinic, she reported newly developed symptoms of an oral ulcer, a sore throat, nausea, vomiting, abdominal pain, diarrhea, polyarthralgia, and multiple swollen joints. Her symptoms began with vomiting and progressed to non-radiating, ongoing, and dull abdominal pain in the umbilical area with diarrhea almost daily as a semisolid. The patient also reported joint pains in the hand, elbows, shoulders, feet, and knees that have worsened with activity.

A review of her 19-month medication history revealed that the patient was on methotrexate at various doses; the most recent dose prescribed was 12.5 mg once weekly, but the patient was taking 10 mg once daily for one-month duration, and accordingly, her symptoms started. The patient was referred to the Emergency Department (ED) for suspicion of methotrexate toxicity. Her vital signs at the ED were as follows: The blood pressure was 94/58 mmHg, the respiratory rate was 18/minute, the pulse rate was 106 beats per minute, and the temperature was 36.5 degrees Celsius. On examination at the ED, she had oral ulcers and tenderness in both the right and left 2nd, 3rd, and 4th metacarpophalangeal bones and warmth in the right extensor carpi ulnaris tenosynovitis. On hand inspection, she had an ulnar deviation on her right hand. A hand tendon examination revealed pain in the extensor digit minima in both hands and the extensor pollicis on both sides. The glenohumeral joint was tender during the shoulder examination. The lower extremity had tenderness in all metatarsal-phalangeal joints and some swelling on the right knee. Laboratory results on ED admission were as follows: (typical values between brackets): Hemoglobin was 11.5 g/dl ( 12-15 g/dl), White Blood Cells (WBC) was 9200/microliter (4000-10000 / microliter), platelet counts were 569 x10^3/ microliter (150-410 x10^3/ microliter), Blood Urea Nitrogen ( BUN) was 4.0 mmol/L (2.5-7.8 mmol/L), creatinine was 46 micromole/L (44-80 micromole/L), Aspartate aminotransferase (AST) was 57 IU/L (0-32 IU/L, Alanine transaminase (ALT) was 50 IU/L (0-33 IU/L), Prothrombin time (PT) was 12.8s (9.4-12.5 seconds), a partial thromboplastin time (PTT) was 29.1 (25.1-36.5 seconds), International Normalized Ratio (INR) was 1.1, The total bilirubin level was 11 micromole/L /L (0–21 micromole/L /L). The baseline methotrexate level was < 0.04 micromole/L.

The patient was started on methotrexate overdose treatment immediately. Leucovorin (17 mg) was initiated via IV. Although the baseline methotrexate level result was very low (< 0.04 micromol/L), a decision was made based on history and symptoms to stop methotrexate and discharge the patient with treatment with oral leucovorin (15 mg every 6 hours for 14 days). After seven days, the patient followed up and reported that the diarrhea had subsided. But she was still experiencing oral pain, ulcers, joint pain, and swelling. The elevated AST and ALT returned to normal. After 14 days of follow-up, the oral ulcer, abdominal pain, and diarrhea subsided, and the leucovorin was discontinued. The patient continued to have rheumatoid arthritis flare, and new options for treatment were discussed.

## Discussions

Chronic methotrexate toxicity is considered more severe than acute methotrexate toxicity.^13^ Although methotrexate levels are considered a guide to the risk of toxicity in patients receiving high doses of methotrexate,^9,13,14^ there is no justification for MTX therapeutic drug monitoring in the case of low-dose toxicity^15,16^ as the pharmacokinetics of methotrexate are highly variable and unpredictable.^17,18^

Chronic toxicity data are limited in the literature. In one retrospective study assessing acute versus chronic methotrexate poisoning, many patients in the study lacked the duration of administration and levels ^(6)^. In one case report, the patient used MTX at a dose of 1.25 mg daily for five consecutive months and 2.5 mg five days a week in the last month^19^ for a total accumulative dose of around 238 mg, but the level was unavailable. In this case, our patient received 10 mg daily for one month (a total accumulative dose of about 300 mg) when she was outside the country, which was discovered when she returned to Qatar. The primary dose-limiting issue for the use of MTX is gastrointestinal toxicity ^(16)^, which should be treated by withdrawal of MTX and delivery of intravenous folinic acid (leucovorin) as soon as possible following exposure.

Most chronic overdoses are due to unintentional methotrexate ingestions because of frequent mistakes.^20^ That’s why methotrexate for non-oncologic use has been included on the Institute for Safe Medication Practices (ISMP) List of High-Alert Medications since the inception of the list in 2003. Although the ISMP recommended best-practice measures, there is still a lack of implementation.^21^ A computerized order entry system is used in our hospital, with a default once-weekly dose for non-oncology indications. Proper education by the clinical pharmacist is conducted for all patients on discharge to ensure understanding and compliance with the prescribed dose to avoid side effects. This case was followed in Qatar, although the wrong dose was initiated when the patient was traveling outside Qatar. Methotrexate levels are not reliable in these chronic unintentional overdose cases, so MTX chronic toxicity still necessitates determining the minimum toxic dose and duration to determine the risk of toxicity.

## Conclusions

MTX dosing errors are one of the causes of MTX toxicity. In a patient who received 10 mg of MTX daily for one month, the serum MTX level was determined to be < 0.04 micromol/L. It was concluded that <0.04 micromol/L MTX in serum can cause toxicity in cases of chronic low-dose toxicity of MTX. Fortunately, our patient was treated after one month of chronic MTX toxicity and recovered within two weeks with folinic acid treatment. Identification of the minimally toxic MTX dose and duration of chronic ingestion can be an area of research to assess the risk of toxicity and correlate it with signs and symptoms. Also, proper counseling and patient education should be implemented to avoid this type of unintentional overdose error.
